# A statistical analysis plan for the Adjunctive Corticosteroids for Tuberculous meningitis in HIV-positive adults (ACT HIV) clinical trial

**DOI:** 10.12688/wellcomeopenres.17154.2

**Published:** 2022-07-07

**Authors:** Joseph Donovan, Trinh Dong Huu Khanh, Guy E. Thwaites, Ronald B. Geskus, ACT HIV investigators

**Affiliations:** 1 Oxford University Clinical Research Unit, Centre for Tropical Medicine, Ho Chi Minh City, Vietnam; 2 Centre for Tropical Medicine and Global Health, Nuffield Department of Medicine, University of Oxford, Oxford, UK; 3 London School of Hygiene and Tropical Medicine, London, UK

**Keywords:** Tuberculous meningitis, human immunodeficiency virus, corticosteroids, clinical trial, analysis plan

## Abstract

TBM is the most severe form of tuberculosis. Clinical trial data are required to provide an evidence base for adjunctive dexamethasone in HIV-positive individuals with TBM, and to guide clinical practice. This document details the planned analyses at 12 months post randomisation for the ACT HIV clinical trial (NCT03092817); ‘a randomised double-blind placebo-controlled trial of adjunctive dexamethasone for the treatment of HIV co-infected adults with tuberculous meningitis (TBM)’. The primary endpoint of the ACT HIV trial is death (from any cause) over the first 12 months after randomisation. This statistical analysis plan expands upon and updates the analysis plan outlined in the published study protocol.

## Scope of document

This document details the planned analyses at 12 months post randomisation for the ACT HIV clinical trial (NCT03092817); ‘a randomised double-blind placebo-controlled trial of adjunctive dexamethasone for the treatment of HIV co-infected adults with tuberculous meningitis (TBM)’. A further analysis will follow at 24 months post randomisation. This statistical analysis plan expands upon and updates the analysis plan outlined in the published study protocol ^1^.

## Background and rationale for study

The ACT HIV clinical trial is a randomised double-blind placebo-controlled trial of adjunctive dexamethasone for the treatment of tuberculous meningitis (TBM) in HIV-positive adults. TBM is the most severe form of tuberculosis, with mortality approaching 50% in people living with HIV ^2– 5^ despite the best available appropriate anti-TB chemotherapy.

TBM develops when *Mycobacterium tuberculosis*, initially acquired via the respiratory route, disseminates via the blood to form a secondary focus in the brain. Rupture of this secondary focus into the subarachnoid space results in TBM, with the consequent host inflammatory response being important for bacterial killing, but also responsible for the pathological complications and often-fatal consequences of the infection.

Adjunctive anti-inflammatory corticosteroid therapy, which may control excessive host inflammation, has long been considered to have a role in TBM treatment ^6^. In 2004, a landmark clinical trial in Vietnam demonstrated that a 6–8 week (duration dependent on Modified Research Council [MRC] TBM severity grade) tapering course of dexamethasone reduced mortality in adults and adolescents with TBM ^4^. In this study, only 98/545 (18.0%) individuals were HIV positive, which was too small to determine if dexamethasone benefited this important sub-group of patients. No other trials have been published since 2004 that examine whether corticosteroids improve outcomes from TBM in HIV-positive adults.

How dexamethasone might confer its clinical benefit on those with TBM remains uncertain. A recent study of cerebrospinal fluid (CSF) cytokines in TBM suggested that HIV-positive individuals have higher cytokine concentrations than HIV-negative individuals ^7^. Whether adjunctive dexamethasone is more or less likely to be beneficial in HIV-positive individuals with TBM is not known. There are an estimated 100,000 cases of TBM globally each year ^8^, many associated with HIV. Clinical trial data are required to provide an evidence base for adjunctive dexamethasone in HIV-positive individuals with TBM, and to guide clinical practice.

## Structure and status of trial

The ACT HIV trial commenced recruitment on 25 ^th^ May 2017. By April 29 ^th^ 2021, the predefined sample size of 520 adults with TBM and HIV co-infection had been enrolled from four hospitals in Vietnam and Indonesia: the Hospital for Tropical Diseases (HTD) and Pham Ngoc Thach Hospital for Tuberculosis and Lung Disease (PNT) in Ho Chi Minh City, Vietnam, and Cipto Mangunkusumo Hospital and Persahabatan Hospital in Jakarta, Indonesia. Detailed enrolment criteria for ACT HIV, including consent and ethical approvals, are described in the published trial protocol ^1^.

Once enrolled, study participants were randomised to dexamethasone or placebo (a double-blinded allocation), with this intervention termed ‘study drug’. Randomisation was stratified by TBM MRC severity score and by hospital. Participants with MRC grade 1 TBM received a 6-week tapering course of study drug, whereas participants with MRC grades 2 or 3 received an 8-week tapering course of study drug. Study drug regimens are shown in Table 1. Participants then underwent clinical assessments at baseline, at days 3, 7, 10, 14, 21, and 30, monthly until month 12, and then 3-monthly until 24 months. Baseline assessment included blood tests, chest X-ray, lumbar puncture, and brain imaging.

**Table 1.** Study drug treatment regimen following randomisation.

	MRC Grade I Daily dexamethasone dose/route	MRC Grades II and III Daily dexamethasone dose/route
Week 1	0.3 mg/kg/24 hrs IV	0.4 mg/kg/24 hrs IV
Week 2	0.2 mg/kg/24 hrs IV	0.3 mg/kg/24 hrs IV
Week 3	0.1 mg/kg/24 hrs IV	0.2 mg/kg/24 hrs IV
Week 4	3mg/24 hrs oral	0.1 mg/kg/24 hrs IV
Week 5	2mg/24 hrs oral	4 mg/24 hrs oral
Week 6	1 mg/24 hrs oral	3 mg/24 hrs oral
Week 7	Stop	2 mg/24 hrs oral
Week 8		1 mg/24 hrs oral

IV=intravenous. MRC=Modified Research Council.

ACT HIV has numerous sub-studies, as described in the protocol ^1^. Key follow up outcomes include survival, disability, and severe adverse events; especially neurological events, events requiring corticosteroids, HIV-associated complications (malignancies, immune reconstitution inflammatory syndrome [IRIS]), and adverse events considered related to dexamethasone.

## Trial endpoints

### Primary endpoint

The primary endpoint of the ACT HIV trial is death (from any cause) over the first 12 months after randomisation.

### Secondary endpoints

The secondary endpoints of the ACT HIV trial are as follows:

1. Neurological disability at 12 months from randomisation

2. First new neurological event or death over the first 12 months after randomisation

3. Neurological IRIS events over the first 6 months after randomisation

4. New AIDS-defining event or death over the first 12 months after randomisation

5. HIV-associated malignancy by 12 months from randomisation

6. Use of open-label corticosteroid treatment for any reason over the first 12 months after randomisation

7. Requirement for shunt surgery by 12 months

8. Any serious adverse events reported by 12 months from randomisation

## Individuals included in analysis

### Primary analysis

The primary analysis includes all randomised participants. As such, an intention-to-treat (ITT) analysis will be performed for the primary and secondary endpoints. Participants will remain included in this analysis even if no study drug was received after randomisation.

### Per protocol analysis

The per protocol (PP) analysis includes all randomised patients with the exception of those subsequently found to have not met all inclusion criteria, or to have met any exclusion criteria, at the time of enrolment, those with a final diagnosis other than TBM (confirmed by microbiology, serology, or histopathology), and those who received less than 7 days of administration of the randomised study drug for reasons other than death, or less than 30 days of anti-tuberculosis drugs for any reason other than death. Trial inclusion and exclusion criteria are listed in the trial protocol ^1^. A per protocol analysis will be performed for all primary and secondary endpoints.

## Statistical software

Data will be analysed using the program R ^9^, using the most up to date version available at the time of final analysis.

## Baseline characteristics

Definition: The following baseline characteristics will be summarised by treatment arm for ITT and PP analyses: age, sex, country, site, diagnostic category (definite, probable, possible, or not TBM by Marais criteria ^10^), history of previous tuberculosis treatment, chest X-ray findings (no TB/miliary TB/pulmonary TB), enrolment MRC TBM grade, enrolment Glasgow coma score (GCS), weight (kg), duration of symptoms, cranial nerve palsy, hemiplegia, para/tetraplegia, urinary retention, history of diabetes, HbA1c, history of intravenous drug use, hepatitis B sAg positivity, hepatitis C Ab positivity, alanine aminotransferase (ALT), bilirubin, full blood count (haemoglobin, white cell count, platelets), plasma sodium, *leukotriene A4 hydrolase* ( *LTA4H*) genotype (CC/CT/TT), routine CSF parameters (opening pressure, total leucocytes, total neutrophils, total lymphocytes, protein, blood:CSF glucose ratio, Ziehl-Neelsen stain, Gene Xpert MTB/RIF, Gene Xpert MTB/RIF Ultra, mycobacterial culture), duration of anti-tuberculosis chemotherapy (days) before enrolment, enrolment anti-TB regimen, anti-TB drug susceptibility results in culture-confirmed sub-group (multi-drug resistant [MDR] TB or rifampicin mono-resistant TB, isoniazid resistant non-MDR, pre-extensively drug resistant [XDR] TB, XDR TB, no or other resistance), HIV infection (new or known diagnosis), anti-retroviral therapy (ART) treatment at enrolment (ART naïve, ≤3 months of ART, >3 months of ART) and time to starting ART (if ART naïve), enrolment CD4 count (median values and categories [<50, 51–100, 101–200, >200]), HIV viral load. These will be described in a baseline variable table ( Table 2).

**Table 2.** Baseline characteristics template table.

Characteristic	Dexamethasone (N=XX)		Placebo (N=XX)	
	N	Summary statistic	N	Summary statistic
Age (years)	XX	XX (XX,XX)	XX	XX (XX,XX)
Sex - male	XX	XX (XX%)	XX	XX (XX%)
Country	XX	XX (XX%)	XX	XX (XX%)
Site - HTD - PNT - Cipto Mangunkusumo - Persahabatan	XX XX XX XX	XX (XX%) XX (XX%) XX (XX%) XX (XX%)	XX XX XX XX	XX (XX%) XX (XX%) XX (XX%) XX (XX%)
Diagnostic category - Definite TBM - Probable TBM - Possible TBM - Not TBM	XX XX XX XX	XX (XX%) XX (XX%) XX (XX%) XX (XX%)	XX XX XX XX	XX (XX%) XX (XX%) XX (XX%) XX (XX%)
Previous tuberculosis treatment	XX	XX (XX%)	XX	XX (XX%)
Chest X-ray findings - No tuberculosis - Miliary tuberculosis - Pulmonary tuberculosis	XX XX XX	XX (XX%) XX (XX%) XX (XX%)	XX XX XX	XX (XX%) XX (XX%) XX (XX%)
Modified MRC grade - Grade I - Grade II - Grade III	XX XX XX	XX (XX%) XX (XX%) XX (XX%)	XX XX XX	XX (XX%) XX (XX%) XX (XX%)
Glasgow coma score	XX	XX (XX,XX)	XX	XX (XX,XX)
Weight (kg)	XX	XX (XX,XX)	XX	XX (XX,XX)
Duration of symptoms (days)	XX	XX (XX,XX)	XX	XX (XX,XX)
Neurological signs - Cranial nerve palsy - Hemiplegia - Paraplegia/tetraplegia - Urinary retention	XX XX XX XX	XX (XX%) XX (XX%) XX (XX%) XX (XX%)	XX XX XX XX	XX (XX%) XX (XX%) XX (XX%) XX (XX%)
History of diabetes	XX	XX (XX%)	XX	XX (XX%)
HbA1c (%)	XX	XX (XX,XX)	XX	XX (XX,XX)
History of intravenous drug use	XX	XX (XX%)	XX	XX (XX%)
Hepatitis B sAg positivity,	XX	XX (XX%)	XX	XX (XX%)
Hepatitis C Ab positivity	XX	XX (XX%)	XX	XX (XX%)
Alanine aminotransferase (ALT) (IU/L)	XX	XX (XX,XX)	XX	XX (XX,XX)
Bilirubin (µmol/L)	XX	XX (XX,XX)	XX	XX (XX,XX)
Full blood count - Haemoglobin (g/dL) - White cell count (x10 ^3^/ µL) - Platelets (x10 ^3^/ µL)	XX XX XX	XX (XX,XX) XX (XX,XX) XX (XX,XX)	XX XX XX	XX (XX,XX) XX (XX,XX) XX (XX,XX)
Plasma sodium (mmol/L)				
*Leukotriene A4 hydrolase* - TT - CT - CC	XX XX XX	XX (XX%) XX (XX%) XX (XX%)	XX XX XX	XX (XX%) XX (XX%) XX (XX%)
CSF parameters - Opening pressure (cmH _2_0) - Total leucocytes (cells/mm ^3^) - Total neutrophils (cells/mm ^3^) - Total lymphocytes (cells/mm ^3^) - Protein (g/L) - Blood:CSF glucose	XX XX XX XX XX XX	XX (XX,XX) XX (XX,XX) XX (XX,XX) XX (XX,XX) XX (XX,XX) XX (XX,XX)	XX XX XX XX XX XX	XX (XX,XX) XX (XX,XX) XX (XX,XX) XX (XX,XX) XX (XX,XX) XX (XX,XX)
CSF microbiological tests - Positive ZN stain - Positive GeneXpert MTB/RIF - Positive GeneXpert MTB/RIF Ultra - Positive mycobacterial culture	XX XX XX XX	XX (XX%) XX (XX%) XX (XX%) XX (XX%)	XX XX XX XX	XX (XX%) XX (XX%) XX (XX%) XX (XX%)
Duration of anti-tuberculosis chemotherapy before enrolment (days)	XX	XX (XX,XX)	XX	XX (XX,XX)
Enrolment anti-tuberculosis chemotherapy regimen - Rifampicin - Isoniazid - Pyrazinamide - Ethambutol - Streptomycin	XX XX XX XX XX	XX (XX%) XX (XX%) XX (XX%) XX (XX%) XX (XX%)	XX XX XX XX XX	XX (XX%) XX (XX%) XX (XX%) XX (XX%) XX (XX%)
Anti-tuberculosis drug resistance * - Multi-drug resistant or rifampicin mono-resistant - Isoniazid resistant non-MDR - Pre-XDR - XDR - No or other resistance	XX XX XX XX XX	XX (XX%) XX (XX%) XX (XX%) XX (XX%) XX (XX%)	XX XX XX XX XX	XX (XX%) XX (XX%) XX (XX%) XX (XX%) XX (XX%)
HIV infection: new diagnosis or known positive status	XX	XX (XX%)	XX	XX (XX%)
ART status at enrolment - ART naïve - ≤3 months of ART - >3 months of ART - Unknown - Undetermined	XX XX XX XX XX	XX (XX%) XX (XX%) XX (XX%) XX (XX%) XX (XX%)	XX XX XX XX XX	XX (XX%) XX (XX%) XX (XX%) XX (XX%) XX (XX%)
Time to starting ART ^#^	XX	XX (XX,XX)	XX	XX (XX,XX)
Enrolment CD4 count (per mm ^3^)	XX	XX (XX,XX)	XX	XX (XX,XX)
Enrolment CD4 count (per mm ^3^) - <50 - 51–100 - 101–200 - >200	XX XX XX XX	XX (XX%) XX (XX%) XX (XX%) XX (XX%)	XX XX XX XX	XX (XX%) XX (XX%) XX (XX%) XX (XX%)
HIV viral load (copies/mL)	XX	XX (XX,XX)	XX	XX (XX,XX)

*Results given for sub-group with positive mycobacterial culture on baseline CSF. Pre-XDR and XDR are defined following World Health Organisation definitions ^11^. ^#^Results given for sub-group naïve to ART at enrolment. N = number of patients included in that statistic. Summary statistic = the median (1 ^st^ and 3 ^rd^ quartile) value for continuous data, and the number and frequency (%) of patients with the characteristic for categorical data. Definite TBM = positive acid fast bacilli (AFB) on CSF Ziehl Neelsen stain, or positive CSF TB GeneXpert test, OR positive CSF TB culture. Probable or possible TBM defined following uniform case defintion ^10^. Confirmed non-TBM = microbiologically confirmed other brain infection. Confirmed additional brain infection includes positive CSF India Ink stain, OR CSF cryptococcal antigen, OR positive blood cryptococcal antigen, OR positive CSF bacterial Gram stain, OR positive CSF bacterial culture, OR positive CSF viral or helminth PCR test. ART status of a patient will be unknown if i) they are on ART treatment and the start ART date is missing completely; or ii) their ART status is unknown. ART status of a patient will be undetermined if start ART date is present but distinction between ≤3 months of ART, and >3 months of ART, cannot be made due to limited date information. ART=antiretroviral therapy. HTD=Hospital for Tropical Diseases. MRC=Modified Research Council. PNT=Pham Ngoc Thach Hospital for Tuberculosis and Lung Disease. TBM=tuberculous meningitis. XDR=Extensively drug resistant. Xpert=Gene Xpert MTB/RIF. ZN=Ziehl Neelsen.

Analysis: Baseline characteristics will be summarised as median (lower and upper quartiles) for continuous data and frequency (percentage) for categorical data. The amount of missing data for each baseline characteristic will also be displayed. We will perform multiple imputation of a variable in case of >5% missing values, and it is assumed that data are missing at random. Otherwise, a complete case analysis will be performed.

### Use of the uniform case definition diagnostic score

The published TBM diagnostic score ^10^ will be used and subjects will be categorised as ‘definite’, ‘probable’, ‘possible’, or ‘not TBM’. Participants will only be categorised as ‘not TBM’ if they have a confirmed alternative diagnosis (alternative to TBM) or they recovered without TB drugs.

## Primary endpoint

The primary endpoint of this trial is death over the first 12 months after randomisation. The primary analysis is a Cox proportional hazards regression model with treatment as the only covariate. Since TBM MRC severity grade at enrolment (I, II, or III) is an important risk factor for mortality and was used as stratification variable in the randomisation, we will additionally perform a secondary analysis with MRC grade added as stratum variable. The proportional hazards assumption will be formally tested based on scaled Schoenfeld residuals and visually assessed by a plot of the scaled Schoenfeld residuals versus transformed time. Deaths, by study drug arm, and the results of the analyses will be shown as per Table 3 and Table 4. 

**Table 3.** Hazard ratio for death (from any cause) over the first 12 months after randomisation.

	No. of deaths		Hazard ratio (95% CI); p value	Test for proportional hazards
	Dexamethasone	Placebo		
All patients Corrected for modified MRC grade	XX/XX	XX/XX	X.XX (X.XX, X.XX) X.XX (X.XX, X.XX)	X.XX X.XX

The primary endpoint will be overall survival, i.e., time from randomisation to death, over the first 12 months of follow-up. This table reports the results from the Cox proportional hazards regression model. The primary effect measure will be the resulting hazard ratio comparing dexamethasone vs. placebo with a corresponding two-sided 95% confidence interval. In "All patients", treatment will be the only covariate. We will additionally report the hazard ratio if the modified MRC grade is included as stratum variable. The test for proportional hazards will use the Kaplan-Meier as time transformation. MRC=Modified Research Council.

**Table 4.** Hazard ratios for death (from any cause) by sub-group over the first 12 months after randomisation.

	No. of deaths		Hazard ratio (95% CI); p value	Test for proportional hazards	p-value for heterogeneity *
	Dexamethasone	Placebo			
Modified MRC grade: - Grade I - Grade II - Grade III	XX/XX XX/XX XX/XX	XX/XX XX/XX XX/XX	X.XX (X.XX, X.XX) X.XX (X.XX, X.XX) X.XX (X.XX, X.XX)	X.XX X.XX X.XX	X.XX
Diagnostic category - Definite TBM - Probable TBM - Possible TBM - Not TBM	XX/XX XX/XX XX/XX XX/XX	XX/XX XX/XX XX/XX XX/XX	X.XX (X.XX, X.XX) X.XX (X.XX, X.XX) X.XX (X.XX, X.XX) X.XX (X.XX, X.XX)	X.XX X.XX X.XX X.XX	X.XX
*Leukotriene A4 hydrolase* - TT - CT - CC	XX/XX XX/XX XX/XX	XX/XX XX/XX XX/XX	X.XX (X.XX, X.XX) X.XX (X.XX, X.XX) X.XX (X.XX, X.XX)	X.XX X.XX X.XX	X.XX
Anti-tuberculosis drug resistance ^#^ - Multi-drug resistant or rifampicin mono-resistant - Isoniazid resistant non-MDR - Pre-XDR - XDR - No or other resistance	XX XX XX XX XX	XX (XX%) XX (XX%) XX (XX%) XX (XX%) XX (XX%)	XX XX XX XX XX	X.XX X.XX X.XX X.XX X.XX	X.XX
ART status at enrolment - ART naïve - ≤3 months of ART - >3 months of ART	XX/XX XX/XX XX/XX	XX/XX XX/XX XX/XX	X.XX (X.XX, X.XX) X.XX (X.XX, X.XX) X.XX (X.XX, X.XX)	X.XX X.XX X.XX	X.XX
Enrolment CD4 count (per mm ^3^) - <50 - 51–100 - 101–200 - >200	XX/XX XX/XX XX/XX XX/XX	XX/XX XX/XX XX/XX XX/XX	X.XX (X.XX, X.XX) X.XX (X.XX, X.XX) X.XX (X.XX, X.XX) X.XX (X.XX, X.XX)	X.XX X.XX X.XX X.XX	X.XX

*Heterogeneity will be tested with a Cox regression model that includes an interaction between treatment effect and subgroup. ^#^Results will be given for sub-group with positive mycobacterial culture on baseline CSF. Pre-XDR and XDR are defined following World Health Organisation definitions ^11^. The primary endpoint will be overall survival, i.e., time from randomisation to death, over the first 12 months of follow-up. This table reports the results from the Cox proportional hazards regression model. The primary effect measure will be the resulting hazard ratio comparing dexamethasone vs. placebo with a corresponding two-sided 95% confidence interval. In these subgroup analyses, a separate Cox model will be fitted for each value of the subgroup. The "Test for heterogeneity" will be based on the likelihood ratio test that includes subgroup as covariate and compares the models with subgroup as main effect only and with subgroup as treatment effect modifier, with TBM MRC severity grade at enrolment (I, II, or III) as covariates. ART=antiretroviral therapy. MRC=Modified Research Council. TBM=tuberculous meningitis. XDR=extensively drug resistant.

Survivors known to be alive at 12 months will be censored at that time-point and subjects who withdrew or were lost to follow-up before 12 months will be censored at the date they were last known to be alive. Subjects who withdrew or were lost to follow-up before 12 months are estimated to be less than 5% of enrolled participants.

The homogeneity of the treatment effect on overall survival across subgroups will be assessed by formal tests of interaction between treatment and the following pre-defined grouping variables: TBM MRC severity grade at enrolment (I, II, or III), diagnostic category (definite, probable, possible), *LTA4H* genotype (CC/CT/TT), drug resistance pattern (MDR TB or rifampicin mono-resistant TB, isoniazid resistant non-MDR, no or other resistance), ART status at enrolment (ART naïve, ≤3 months of ART, >3 months of ART), and CD4 cell count at enrolment (<50, 51–100, 101–200, >200). We will estimate and report the treatment effect by fitting a separate Cox model per subgroup. We will additionally fit a Cox model that includes CD4 count at enrollment as a continuous variable via a restricted cubic spline with four knots and an interaction term with treatment arm.

Kaplan-Meier plots and explicit survival estimates at 3, 6, and 12 months of follow-up will be calculated for the full sample and in the subgroups defined above. We will compare the treatment arms via the absolute risk of death at 12 months (using a Wald-type test based on Kaplan-Meier estimates at 12 months and associated standard errors using Greenwood’s formula) and the restricted mean survival until 12 months (using the survRM2 ^12^ package in R).

## Secondary endpoints

Secondary outcomes 2 to 7 below are time-to-event outcomes. For each, we will compute and plot the Kaplan-Meier estimates (outcomes 2, 4) or the competing risks Aalen-Johansen estimates (outcomes 3, 5, 6 and 7) and report the values of the estimates at 3, 6 and 12 months. We will also fit a Cox proportional hazards model (results are interpreted as relative cause-specific hazards in the presence of competing risks). Analyses will be performed for the full samples (ITT and PP). Subgroup analyses are specified below per secondary endpoint. For the subgroup analyses, we follow the same procedure as for the primary outcome: we fit separate models per subgroup and we fit a model in which we test for interaction by subgroup.

### 1. Neurological disability at 12 months from randomisation

Neurological disability will be assessed by the modified Rankin score at months 1, 2, 3, 6, 9, and 12 from randomisation ( Table 5). The main secondary endpoint is the 12-month assessment and subjects who died before 12 months will be treated as having a score of 6 (‘dead’). Rankin score assessments will be included from clinical assessments performed monthly (+/- 7 days), with the exception of the 12-month clinical assessment for which an acceptable range of -10 days/+1 month will be applied. All clinical assessment timings are based on days from randomisation (i.e., ‘day 0’ is labeled as the first day study drug is received, with study drug received immediately after randomisation).

**Table 5.** The Modified Rankin Scale.

Score	Description
0	No symptoms
1	Minor symptoms not interfering with lifestyle
2	Symptoms that lead to some restriction in lifestyle, but do not interfere with the patients ability to look after themselves
3	Symptoms that restrict lifestyle and prevent totally independent living
4	Symptoms that clearly prevent independent living, although the patient does not need constant care and attention.
5	Totally dependent, requiring constant help day and night.
6	Death

Neurological disability (as assessed by the ordinal modified Rankin scale) at 12 months will be compared between the two arms with a proportional odds logistic regression model with the treatment assignment as the main covariate and adjustment for TBM MRC severity grade. The result will be summarised as a cumulative odds ratio with corresponding 95% confidence interval and p‐value. Individuals who withdrew or were lost to follow-up before 12 months are excluded.

### 2. First new neurological event or death over the first 12 months after randomisation

A new neurological event is defined as a fall in GCS by ≥2 points for ≥2 days from the highest previously recorded GCS (including baseline) or the onset of any of the following clinical adverse events: cerebellar symptoms, focal neurological signs, or onset of seizures. A description of all the events will be given, summarising how they met the criteria.

Analyses will additionally be performed in the subgroups defined by TBM MRC severity grade, diagnostic category, ART status at enrolment, time from randomisation to start of ART, and baseline CD4 count.

### 3. First IRIS event over the first 6 months after randomisation

The criteria for neurological IRIS diagnosis is defined in the study protocol ^1^. The neurological IRIS rate will be defined as the number of IRIS events divided by the observed person-time of follow-up in each treatment group. A description of all the IRIS events will be given, describing how they met the IRIS diagnostic criteria.

The number of IRIS events in each group will be summarised. Death will be interpreted as a competing risk. Analyses will additionally be performed in the subgroups defined by TBM MRC grade, diagnostic category, ART status at enrolment, time from randomisation to start of ART, and baseline CD4 count.

### 4. AIDS-defining event or death over the first 12 months after randomisation

Acquired immunodeficiency syndrome (AIDS)-defining illnesses will be defined as per the World Health Organisation (WHO) classification. WHO define AIDS as a clinical diagnosis (presumptive or definitive) of any stage 4 HIV condition ^13^.

### 5. HIV-associated malignancy over the first 12 months after randomisation

HIV-associated malignancy is defined as new diagnosis of one or more of the three major HIV-associated malignancies; Kaposi sarcoma, high grade B-cell non-Hodgkin lymphoma or invasive cervical cancer.

The number of HIV-associated malignancy events in each group will be summarised. Death will be interpreted as a competing risk. Analyses will additionally be performed in the subgroups defined by ART status at enrolment.

### 6. Use of open-label corticosteroid treatment for any reason, and at any time, after randomisation

The number of open-label corticosteroid treatment events in each group will be summarised. The reasons for corticosteroid treatment will be listed. Death will be interpreted as a competing risk.

### 7. Requirement for shunt surgery by 12 months

The number of shunts performed in each group will be summarised. Death will be interpreted as a competing risk.

### Adverse events until 12 months

Serious adverse events (SAE) are defined in the study protocol ^1^. SAE will be sub-grouped into categories. SAE will be grouped and graded as per Common Terminology Criteria for Adverse Events (CTCAE) ^14^.

The number of patients with any serious adverse event will be summarised and compared between the two treatment arms based on the chi-squared test, or Fisher’s exact test in case the expected count under the null hypothesis in at least one of the cells is smaller than one ^15^. Specific adverse events will be summarised, but not formally compared. The total number of serious adverse event episodes per patient will also be summarised and informally compared based on a quasi-Poisson regression model with treatment as the only covariate.

The following subgroups of adverse events will also be separately summarised: clinical grade 3&4 adverse events; serious adverse events possibly, probably, or definitely related to the study drug; adverse events leading to TB treatment or ARV interruptions. Grade 3&4 laboratory abnormalities will be summarised in the same way as clinical adverse events. Adverse events will be shown as per Table 6– Table10.

**Table 6a.** Summary of serious adverse events.

Characteristic	Dexamethasone (N=XX)		Placebo (N=XX)		Comparison (p-value)
	N.pt	N.ae	N.pt	N.ae	
Any selected serious adverse event	XX (XX%)	XX	XX (XX%)	XX	X.XX
Serious adverse events of all types	XX (XX%)	XX	XX (XX%)	XX	X.XX

N.pt = the number of patients with at least one serious adverse event (% of all patients receiving the same intervention) N.ae = the total number of episodes of that particular serious adverse event

**Table 6b.** Summary of serious adverse events, shown by reasons for which they were considered serious, not shown by study arm.

	Causes death (N=XX)	Life threatening event * (N=XX)	Hospitalisation of prolongation of hospitalisation (N=XX)	Persistent or significant disability/ incapacity ** (N=XX)	Congenital anomaly/ birth defect (N=XX)	Important medical event which may jeopardise the patient and/or require intervention (N=XX)
	n Summary statistic	n Summary statistic	n Summary statistic	n Summary statistic	n Summary statistic	n Summary statistic
Name of event	XX (XX%)	XX (XX%)	XX (XX%)	XX (XX%)	XX (XX%)	XX (XX%)

* Subjects were at immediate risk of death at the time of the event; it does not refer to an event which hypothetically might have caused death if it were more severe ** A substantial disruption of a person's ability to conduct normal life functions N is the number of all patients, n is the number of patients with a non-missing value Summary statistic is absolute count (%) for categorical variable(s).

**Table 7.** Summary of serious adverse events possibly, probably, or definitely related to the study drug.

Characteristic	Dexamethasone (N=XX)		Placebo (N=XX)		Comparison (p-value)
	N.pt	N.ae	N.pt	N.ae	
Any selected serious adverse event possibly, probably, or definitely related to the study drug	XX (XX%)	XX	XX (XX%)	XX	X.XX
Serious adverse events of all types possibly, probably, or definitely related to the study drug	XX (XX%)	XX	XX (XX%)	XX	X.XX

N.pt = the number of patients with at least one serious adverse event (% of all patients receiving the same intervention) N.ae = the total number of episodes of that particular serious adverse event

**Table 8.** Summary of clinical grade 3&4 adverse events.

Characteristic	Dexamethasone (N=XX)		Placebo (N=XX)		Comparison (p-value)
	N.pt	N.ae	N.pt	N.ae	
Any selected clinical grade 3&4 adverse event	XX (XX%)	XX	XX (XX%)	XX	X.XX
Clinical grade 3 adverse events of all types	XX (XX%)	XX	XX (XX%)	XX	X.XX
Clinical grade 4 adverse events of all types	XX (XX%)	XX	XX (XX%)	XX	X.XX
Clinical grade 3&4 adverse events of all types	XX (XX%)	XX	XX (XX%)	XX	X.XX

N.pt = the number of patients with at least one adverse event (% of all patients receiving the same intervention) N.ae = the total number of episodes of that particular adverse event The ‘clinical grade 3&4 adverse events of all types’ grouping will include only the group of adverse events that cannot be separated into grades 3 or 4 (recorded as ‘grade 3 or 4’ at the beginning of the trial). These events will not also be represented in the above ‘grade 3’ and ‘grade 4’ rows.

**Table 9.** Summary of adverse events leading to TB treatment or antiretroviral (ARV) interruptions.

Characteristic	Dexamethasone (N=XX)		Placebo (N=XX)		Comparison (p-value)
	N.pt	N.ae	N.pt	N.ae	
Any selected adverse event leading to TB treatment or antiretroviral interruptions	XX (XX%)	XX	XX (XX%)	XX	X.XX
Adverse events of all types leading to TB treatment or antiretroviral interruptions	XX (XX%)	XX	XX (XX%)	XX	X.XX

N.pt = the number of patients with at least one serious adverse event (% of all patients receiving the same intervention) N.ae = the total number of episodes of that particular serious adverse event

**Table 10.** Summary of Grade 3&4 laboratory abnormalities.

Characteristic	Dexamethasone (N=XX)		Placebo (N=XX)		Comparison (p-value)
	N.pt	N.ae	N.pt	N.ae	
Any selected grade 3&4 laboratory abnormalities	XX (XX%)	XX	XX (XX%)	XX	X.XX
Grade 3&4 laboratory abnormalities of all types	XX (XX%)	XX	XX (XX%)	XX	X.XX

N.pt = the number of patients with at least one serious adverse event (% of all patients receiving the same intervention) N.ae = the total number of episodes of that particular serious adverse event

### Explanatory analysis via multivariate model

A multivariate model of variables independently associated with death will include: age, enrolment GCS, TBM diagnostic category (definite, probable, possible), study drug allocation (dexamethasone vs. placebo), CD4 count, CSF total leucocytes, CSF total neutrophils, CSF blood:glucose ratio, CSF lactate. Numeric variables will be analysed using restricted cubic splines. We will not include any interactions.

## Data availability

No data is associated with this article.
