# Supplementary material for: A statistical analysis plan for the Adjunctive Corticosteroids for Tuberculous meningitis in HIV-positive adults (ACT HIV) clinical trial
Source: Wellcome Open Res. 2022 Jul 7;6:280. Originally published 2021 Oct 19. [Version 2] doi: 10.12688/wellcomeopenres.17154.2 (PMC8561610; doi:10.12688/wellcomeopenres.17154.2)
Supplement: Peer review history

# Peer review: Reviewer response for version 2

**DOI:** 10.21956/wellcomeopenres.19988.r51397

**Published:** 2022-07-07

**Authors:** Nico (Nicolaas) Nagelkerke

**Affiliations:** 1 Centre for Global Health Research, Unity Health Toronto, Dalla Lana School of Public Health, University of Toronto, Toronto, ON, Canada

Change is just a useful clarification.

Is the study design appropriate for the research question?

Yes

Is the rationale for, and objectives of, the study clearly described?

Yes

Are sufficient details of the methods provided to allow replication by others?

Yes

Are the datasets clearly presented in a useable and accessible format?

Not applicable

Reviewer Expertise:

Biostatistics and Infectious disease epidemiology

I confirm that I have read this submission and believe that I have an appropriate level of expertise to confirm that it is of an acceptable scientific standard.

# Peer review: Reviewer response for version 1

**DOI:** 10.21956/wellcomeopenres.18947.r46542

**Published:** 2021-11-10

**Authors:** Peter Mwaba

**Affiliations:** 1 Faculty of Medicine, Lusaka Apex Medical University, Lusaka, Zambia

Evidence from Clinical trials that have been done this far on disseminated tuberculosis and in Particular TBM have not been conclusive on the role of Corticosteroids in improving outcomes. Corticosteroids have been tried in Tuberculous pericarditis, granulomatous diseases and Leprosy reactions with good outcomes. Their role in bacterial meningitis particularly Waterhouse-Friderichsen Syndrome has been shown to be positive.

 Given the debate on BCG vaccine and its possible role in preventing Tuberculous meningitis it would be of value if there is a specific way of confirming the number of participants who have had the vaccine. Some of the Secondary end points may be drug related and it is therefore important to know the exact drug regimen used for both TBM and HIV. Otherwise an excellent piece of work

Is the study design appropriate for the research question?

Yes

Is the rationale for, and objectives of, the study clearly described?

Yes

Are sufficient details of the methods provided to allow replication by others?

Yes

Are the datasets clearly presented in a useable and accessible format?

Yes

Reviewer Expertise:

Infectious disease

I confirm that I have read this submission and believe that I have an appropriate level of expertise to confirm that it is of an acceptable scientific standard.

# Peer review: Reviewer response for version 1

**DOI:** 10.21956/wellcomeopenres.18947.r46541

**Published:** 2021-11-01

**Authors:** Ravindra Kumar Garg, Hardeep Singh Malhotra

**Affiliations:** 1 Department of Neurology, King George's Medical University, Lucknow, Uttar Pradesh, India; 2 Department of Neurology, King George’s Medical University, Lucknow, Uttar Pradesh, India

The issue of potential benefits of corticosteroids in HIV-associated TBM remains unaddressed so far. The authors have planned an almost flawless study to address the issue. The authors are trying the same corticosteroids regimen as had been used in earlier trials which included both HIV-negative and HIV-positive TBM patients. A small paragraph detailing the anti-tuberculosis regimen (drugs and duration) and the ART regimen should be added for better clarity. How will the authors address the issue of "paradoxical reaction"? I find vision loss a major disability among TBM survivors, kindly give due attention to this as well. 

 Regarding analysis, I feel that the use of median should depend on the distribution instead of assuming an asymmetric distribution. We can, therefore, include 'mean' too and look for its statistical relevance.

 In my opinion, the corticosteroids regimen that has been planned to use in this study is a bit complicated. With a simpler regimen compliance would be better.

Is the study design appropriate for the research question?

Yes

Is the rationale for, and objectives of, the study clearly described?

Yes

Are sufficient details of the methods provided to allow replication by others?

Yes

Are the datasets clearly presented in a useable and accessible format?

Yes

Reviewer Expertise:

CNS tuberculosis and other CNS infections

We confirm that we have read this submission and believe that we have an appropriate level of expertise to confirm that it is of an acceptable scientific standard.

# Peer review: Reviewer response for version 1

**DOI:** 10.21956/wellcomeopenres.18947.r46545

**Published:** 2021-10-27

**Authors:** Nico (Nicolaas) Nagelkerke

**Affiliations:** 1 Centre for Global Health Research, Unity Health Toronto, Dalla Lana School of Public Health, University of Toronto, Toronto, ON, Canada

This is slick analysis plan by a clearly highly experienced team for a multi-centre randomized clinical trial following highly standardized methodologies. The proposed analyses will lead to a correct, and generally accepted result, as well as guide the future standard of care guidelines for Tuberculous meningitis in HIV+ patients. As the drug on trial is cheap and easy to administer, guidelines will be applicable globally (including Africa where HIV prevalence is highest).

Is the study design appropriate for the research question?

Yes

Is the rationale for, and objectives of, the study clearly described?

Yes

Are sufficient details of the methods provided to allow replication by others?

Yes

Are the datasets clearly presented in a useable and accessible format?

Not applicable

Reviewer Expertise:

Biostatistics and Infectious disease epidemiology

I confirm that I have read this submission and believe that I have an appropriate level of expertise to confirm that it is of an acceptable scientific standard.
